# A review of the genus *Pelodiaetus* Jeannel (Coleoptera, Carabidae, Anillini) of New Zealand, with re-description of the genus, description of a new species, and notes on the evolutionary history

**DOI:** 10.3897/zookeys.879.37684

**Published:** 2019-10-09

**Authors:** Igor M. Sokolov

**Affiliations:** 1 Systematic Entomology Laboratory, ARS, USDA, c/o Smithsonian Institution, P.O. Box 37012, National Museum of Natural History, Washington, DC 20013-7012, USA National Museum of Natural History, Smithsonian Institution Washington United States of America

**Keywords:** Adephaga, biogeography, East Gondwana, identification key, new synonym, *Pelodiaetus*, syntopic co-occurrence

## Abstract

On the basis of new morphological data a re-description of the genus *Pelodiaetus* is provided, a new species of the genus *P.nunni***sp. nov.** (Christchurch, Canterbury, South Island) is described, and *P.lewisi* Jeannel is proposed as a synonym of *P.sulcatipennis* Jeannel, **syn. nov.** A taxonomic key as well as distribution maps for species of *Pelodiaetus* are provided. Data on comparative morphology and biogeographical aspects of speciation in the genus *Pelodiaetus* and its morphological relatives from Australia and New Zealand are discussed.

## Introduction

The genus *Pelodiaetus* was erected by Jeannel (1937) for tiny New Zealand anillines from the southern part of the South Island, characterized by the reduced number of setae (= eight) in the elytral series of umbilicate marginal pores, only one (posterior) elytral discal seta, and a distinct elytral longitudinal groove (Jeannel 1937). The genus is composed of two species, *P.sulcatipennis* Jeannel and *P.lewisi* Jeannel, differing from each other in the details of their habitus, mainly in the shape of the pronotum. Later, in his main monograph, Jeannel (1963) recalculated the number of setae in the elytral series of umbilicate marginal pores and stated them as nine, a typical number for the majority of representatives of Anillini, thus rejecting the reduced state of this character. Meanwhile, the last reviser of the New Zealand anillines (Moore 1980) continued to consider the number of pores in the umbilicate series of elytra as incomplete with the seventh and eighth pores “obsolete or obsolescent,” and used this feature for distinguishing representatives of *Pelodiaetus* Jeannel from those of *Pelodiaetodes* Moore in his key for New Zealand anillines. In the same publication, Moore expressed doubts that the genus included two species and pointed out that “the two names may merely represent two extremes of a single species.”

The author had the opportunity to investigate the material on Anillini from the New Zealand Arthropod Collection and the private collection of JT Nunn, whose numerous collecting methods included a soil-washing technique that greatly enriched the number of subterranean species available for study. Preliminary sorting of the material of *Pelodiaetus* showed that this genus includes one undescribed species and one species that needs to be synonymized. In addition, thorough examination of elytral chaetotaxy revealed the discrepancy between the state of this character in the works of previous authors and its actual configuration. Altogether, the description of a new species and corrected re-description of the genus, along with comments on the status of described forms, serves as a basis for this paper.

## Materials and methods

This study is based on the examination of 83 specimens of *Pelodiaetus* from the New Zealand Arthropod Collection (**NZAC**), Auckland, New Zealand, and from the personal collection of John T Nunn (**JTN**), Dunedin, New Zealand. Verbatim label data are given for type specimens of all newly described taxa, with label breaks indicated by a slash (“/”).

### Measurements

All specimens were measured electronically using a Leica M420 microscope equipped with a Syncroscopy AutoMontage Photomicroscopy system (SYNCROSCOPY, Synoptics Ltd.). Measurements for various body parts, given in mm, are encoded as follows:

**LH** length of head, measured along midline from anterior margin of labrum to the virtual line connecting posterior supraorbital setae;

**WH** width of head, at level of anterior supraorbital setae;

**WPm** maximal width across pronotum;

**WPa** width across anterior angles of pronotum;

**WPp** width across posterior angles of pronotum;

**LP** length of pronotum from base to apex along midline;

**WE** width of elytra, at level of 4^th^ umbilicate setae;

**LE** length of the elytra, from apex of scutellum to apex of left elytron;

**SBL** standardized body length, a sum of LH, LP, and LE.

In addition, nine ratios between these measurements were calculated: WH/WPm, WPm/WE, WPa/WPp, WPm/WPp, WPm/LP, WE/SBL, WE/LE, LE/SBL, and LP/LE. All values are given as mean ± standard deviation.

### Illustrations

Digital photographs of the dorsal habitus of new species were taken with the AutoMontage system using a Leica M420 microscope. Line drawings of selected body parts were made using a camera lucida on an Olympus BX 50 microscope. Scanning electron micrographs were made with coating on an ESEM FEI Quanta 200.

### Dissections

Dissections were made using standard techniques. Genitalia were dissected from the abdomens of specimens previously softened in boiling water for 20–30 minutes. Contents of the abdomen were cleared using boiling 10% KOH for 2–3 minutes to remove internal tissues, and then washed in hot water before examination. After examination, genitalia were mounted on plastic transparent boards in dimethylhydantoin formaldehyde resin (DMHF, Steedman 1958) and pinned beneath the specimen. In some species, investigation of body parts was undertaken as follows. The whole specimen was cleared, using boiling 10% KOH for ~5 minutes, then washed and dissected. Disassembled body parts from one specimen were placed on a plastic transparent board, properly oriented, mounted in DMHF and pinned together with the specimen labels.

### Type material

The author had no opportunity to investigate the type material of the New Zealand anillines. The concept of *Pelodiaetus* used in the paper is based on the material identified by Moore during his work on New Zealand fauna of Anillina (Moore 1980).

### Species ranking

Criteria for recognizing new species were the following (Sokolov et al. 2004): two or more similar forms that are sympatric were considered separate species if they differed in genitalic morphology and at least one external character; allopatric forms that were similar in external morphology were considered separate species if they differed in general form of the median lobe or the armature of the internal sac; allopatric forms were considered conspecific if they show intergradation of external characters and/or intergradation of the shape and armature of the median lobe. Therefore, morphological recognition of species was based on gross external characters, including forebody proportions, and form and structure of the elytra, and fine external characters, such as chaetotaxy of different body parts, structure of the labio-maxillar complex, shape of ventral sclerites, and patterns of forebody microsculpture. Genitalic characters included the form of the median lobe, the armature of the internal sac observed retracted inside the median lobe, the shape of spermatheca, and the structure of ovipositor sclerites.

### Terms and descriptions

The terms used in the paper and the scheme of descriptions follow that of the author’s previous publications on New Zealand Anillini (Sokolov 2015, 2016). Numbering of the umbilicate pores of elytra follows that of Erwin (1974) and Giachino and Vailati (2011).

## Taxonomic treatment

### Order Coleoptera Linnaeus, 1758

#### Family Carabidae Latreille, 1802

**Subfamily Trechinae Bonelli, 1810**

**Tribe Anillini Jeannel, 1937**

##### Pelodiaetus

Taxon classificationAnimaliaColeopteraCarabidae

Jeannel, 1937

84D68944-C436-5AC9-AC61-4ACF0A5EB849

Pelodiaetus Jeannel, 1937: 275 (type species Pelodiaetussulcatipennis Jeannel, 1937, by original designation).

###### Recognition.

The members of this genus are distinguished from the other New Zealand representatives of Anillina by the following combination of characters: eyes absent; head with long fronto-lateral carinae; antennae submoniliform, of moderate length; prosternal process slightly dilating to the blunt apex; pronotum cordiform, with prominent anterior angles and with short basal constriction anterior to the projected posterior angles; elytra with oblique longitudinal grooves; elytral apices slightly dehiscent with narrowly rounded sutural angles; pygidium exposed in apical half; 1^st^ elytral discal seta indistinct, only slightly longer then surrounding vestiture, while 2^nd^ and 3^d^ discal setae always clearly visible; elytral margin with umbilicate series of 9 pores: the longest setae in the 2^nd^, 6^th^, and 9^th^ pore positions, 7^th^, 8^th^, and 9^th^ pores equidistant, not aligned, virtually forming obtuse isosceles triangle with 8^th^ pore shifted towards disc. The developed fronto-lateral carinae, antennae of moderate length, projected pronotal anterior angles, grooved elytra, exposed pygidium and small size separate *Pelodiaetus* from endogean *Hygranillus* Moore. Distinct posterior angles of pronotum, dilated prosternal process and grooved elytra distinguish the representatives of *Pelodiaetus* from the species of *Zeanillus* Jeannel. Umbilicate series of nine pores, slightly dehiscent apices of grooved elytra and exposed pygidium separate the members of *Pelodiaetus* from the species of *Nesamblyops* Jeannel. An absence of the distinct tubercle anterior to the posterior angles of the pronotum and the small size separate the species of *Pelodiaetus* from the species of *Pelodiaetodes* Moore.

###### Description.

***Size.***SBL range 1.19–1.45 mm.

***Habitus.*** Body form slightly convex, almost subparallel (Fig. 6), moderately elongate (WE/SBL range 0.31–0.35), head relatively wide, subequal to the width of pronotum (WH/WPm range 0.80–0.85), pronotum relatively wide, subequal to the width of elytra (WPm/WE range 0.79–0.88).

***Color.*** Body rufo-testaceous or testaceous, appendages testaceous.

***Microsculpture.*** Dorsal microsculpture of polygonal sculpticells with isodiametric mesh pattern throughout the dorsal surface. Development of microsculpture varies on different body parts. Head and disc of pronotum with shallow microlines, sometimes partially obliterated, while on elytra microlines are very distinct, forming well-pronounced sculpticells.

***Luster.*** Body surface shiny.

***Macrosculpture.*** Body surface sparsely and finely punctate.

***Vestiture.*** Body surface covered with sparse yellowish short setae. Vestiture of elytra moderately long (around one-half length of discal setae).

**Figure 1.**
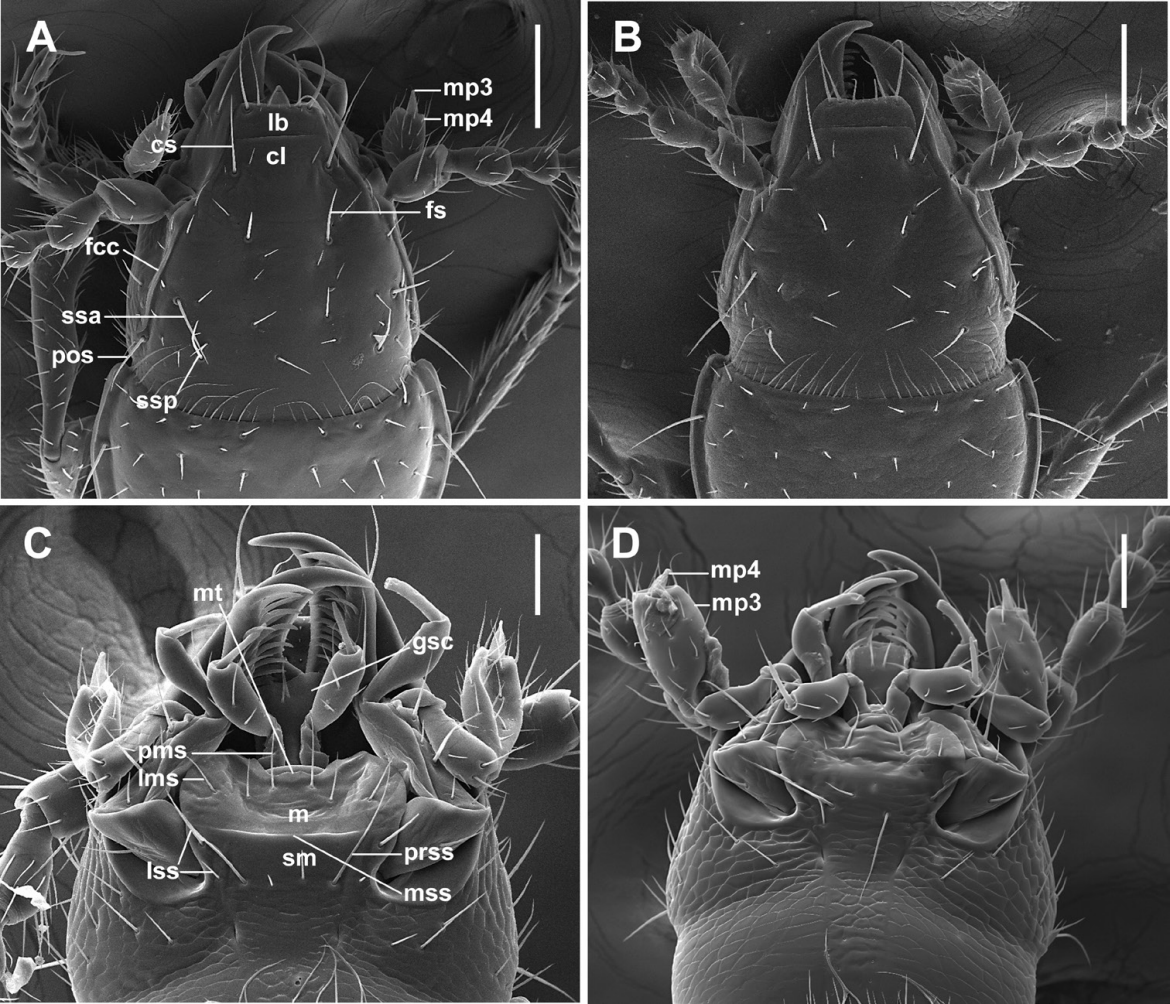
SEM illustrations of head, dorsal aspect, and labial complex, ventral aspect, of *Pelodiaetus* species. **A, C***P.sulcatipennis***B, D***P.nunni*. Abbreviations: cl – clypeus; cs – clypeal seta; fcc – fronto-clypeal carina; fs – frontal seta; gsc – glossal sclerite; lb – labrum; lms – lateral mental seta; lss – lateral submental setae; m – mentum; mp3 – maxillary palpomere 3; mp4 – maxillary palpomere 4; mt – mental tooth; mss – mental-submental suture; pms – paramedial mental seta; pos – postorbital seta; prss – primary basal submental seta; sm – submentum; ssa – anterior supraorbital seta; ssp – posterior supraorbital seta. Scale bars: 0.1 mm (**A, B**); 0.05 mm (**C, D**).

**Figure 2.**
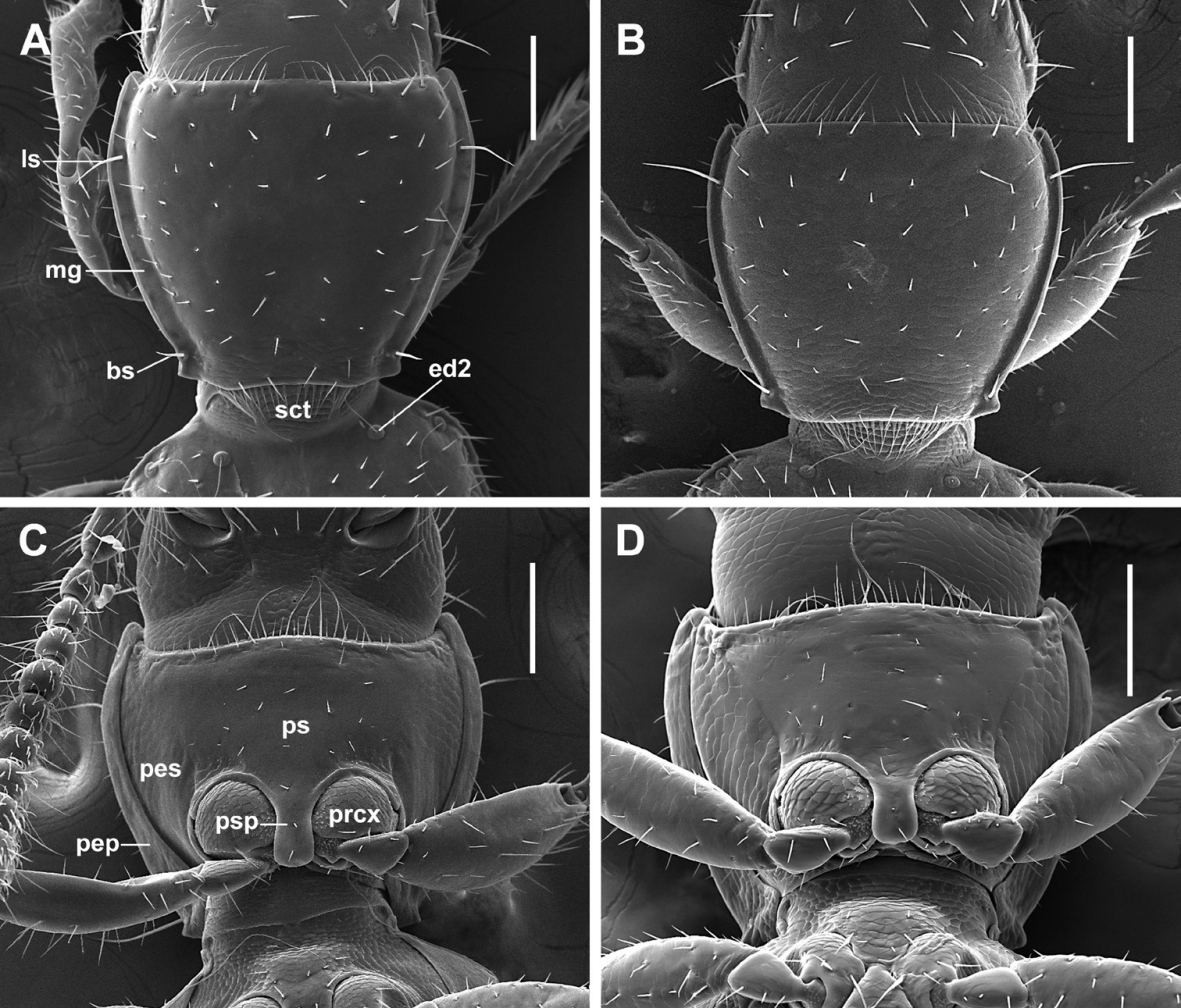
SEM illustrations of pronotum, dorsal aspect, and prothorax, ventral aspect, of *Pelodiaetus* species. **A, C***P.sulcatipennis***B, D***P.nunni*. Abbreviations: bs – basilateral pronotal seta; ed2 – scutellar seta; ls – midlateral pronotal seta; mg – marginal pronotal gutter; pep – proepipleuron; pes – proepisternum; prcx – procoxa; ps – prosternum; psp – prosternal intercoxal process; sct – scutellum. Scale bars 0.1 mm.

***Fixed setae.*** Primary head setae include a pair of clypeal (cs), a pair of frontal (fs), two pairs of supraorbital (ssa and ssp) and one pair of postorbital (pos) setae (Fig. 1A, B). Mentum with two pairs of long primary (paramedial and lateral) setae (Fig. 1C, D, pms, lms). Submentum with three pairs of long primary setae (lss, prss) and a few additional shorter setulae (Fig. 1C, D). Pronotum with two long primary lateral setae (midlateral, ls, and basilateral, bs) on each side (Fig. 2A, B). Elytra with two distinct discal setae (Fig. 4, ed5 and ed6), first discal seta (ed3) barely visible, scutellar (ed2) and apical (ed8) setae of normal sizes. Last three (7^th^, 8^th^, and 9^th^) pores (eo7, eo8, and eo9) of umbilicate series equidistant, not aligned, with 8^th^ pore shifted towards the disc and virtually forming an obtuse isosceles triangle, the longest setae of umbilicate series in the 2^nd^, 6^th^, and 9^th^ pore positions (Fig. 4). Fifth visible sternite of male with two and of female with four setae along the posterior margin.

***Head*** (Fig. 1A, B). Anterior margin of clypeus (cl) straight. Frontal area flat without tubercle medially near frontoclypeal suture. Fronto-lateral carinae distinct and long (fcc).

***Eyes.*** Eyes absent.

***Antennae.*** Submoniliform, 11-segmented, extended to about posterior margin of pronotum. Antennomeres 1 and 2 elongate, of equal length and 1.3–1.4 times longer than antennomere 3, which is slightly elongate and 1.1 times longer than antennomere 4. Antennomeres 4-10 globose, last antennomere conical and 1.7–1.8 times longer than penultimate antennomere.

***Labrum*** (Fig. 1A, B). Labrum (lb) transverse with almost straight, entire anterior margin with six setae apically, increasing in size from the central pair outwards.

***Labium*** (Fig. 1C, D). Labium with almost obliterated blunt mental tooth (mt); mentum (m) and submentum (sm) split, with mental-submental suture (mss). Glossal sclerite (gsc) without paraglossae, bisetose.

***Prothorax.*** Pronotum (Fig. 2A, B) cordiform, slightly convex, arcuately constricted posteriorly and moderately sinuated anterior to posterior angles, with wide marginal gutter (mg). Posterior margin of pronotum shallowly concave medially and oblique laterally. Anterior angles narrowly rounded, distinctly projecting forward. Posterior angles rectangular, projecting outwards, bearing basolateral seta anterior to angles. Widths across anterior margin slightly to moderately greater than between posterior angles at the level of basilateral setae (WPa/WPp range 1.11–1.40). Prosternum (Fig. 2C, D) slightly protruding at the anterior margin medially, there with a group of longer setae relative to other prosternal vestiture. Prosternal intercoxal process (psp) unmargined, slightly dilated apically and widely rounded at apex.

***Scutellum*** (Fig. 2A, B). Externally visible, triangular, with rounded apex.

***Elytra*** (Fig. 4). Elytra subdepressed, relatively long (LE/SBL from 0.56 to 0.60 among specimens) with oblique longitudinal grooves. Humeri rounded, forming an oblique angle with the longitudinal axis of body. Elytral basal margination lacking (Fig. 2A, B). Apical half of elytra without subapical sinuation. Sutural angle of elytron narrowly rounded, making apices of elytra slightly dehiscent (Fig. 4).

***Hind wings.*** Absent.

**Figure 3.**
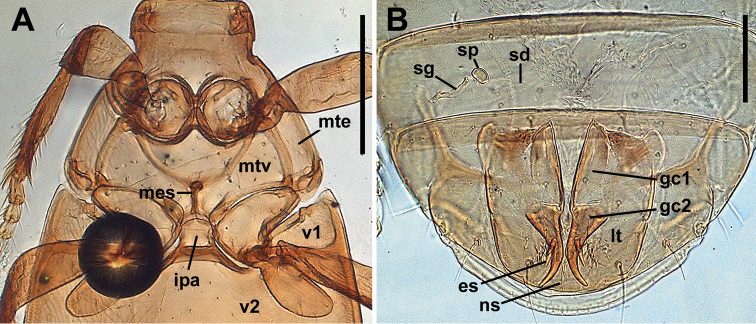
Digital images of pterothorax of *Pelodiaetussulcatipennis* (**A**) and last abdominal ventrites with female genitalia (**B**), ventral aspect. Abbreviations: ipa – intercoxal process of abdominal ventrite 2; mes – metendosternite; mte – metanepisternum; mtv – metaventrite; v1-v2 – abdominal ventrites 1-2; es – ensiform seta; gc1 – gonocoxite 1; gc2 – gonocoxite 2; lt – laterotergite; ns – nematiform seta; sd – spermathecal duct; sg – spermathecal gland; sp – spermatheca. Scale bars: 200 µm (**A**); 100 µm (**B**).

***Pterothorax*** (Fig. 3A). Metaventrite (mtv) moderately short, distance between meso- and metacoxae approximately equals diameter of mesocoxa. Metanepisternum (mte) slightly elongate, rectangle, with anterior margin shorter than outer margin. Metendoventrite (mes) with reduced anterior part and with lateral arms. Lateral arms U-shaped with widely divergent branches.

**Figure 4.**
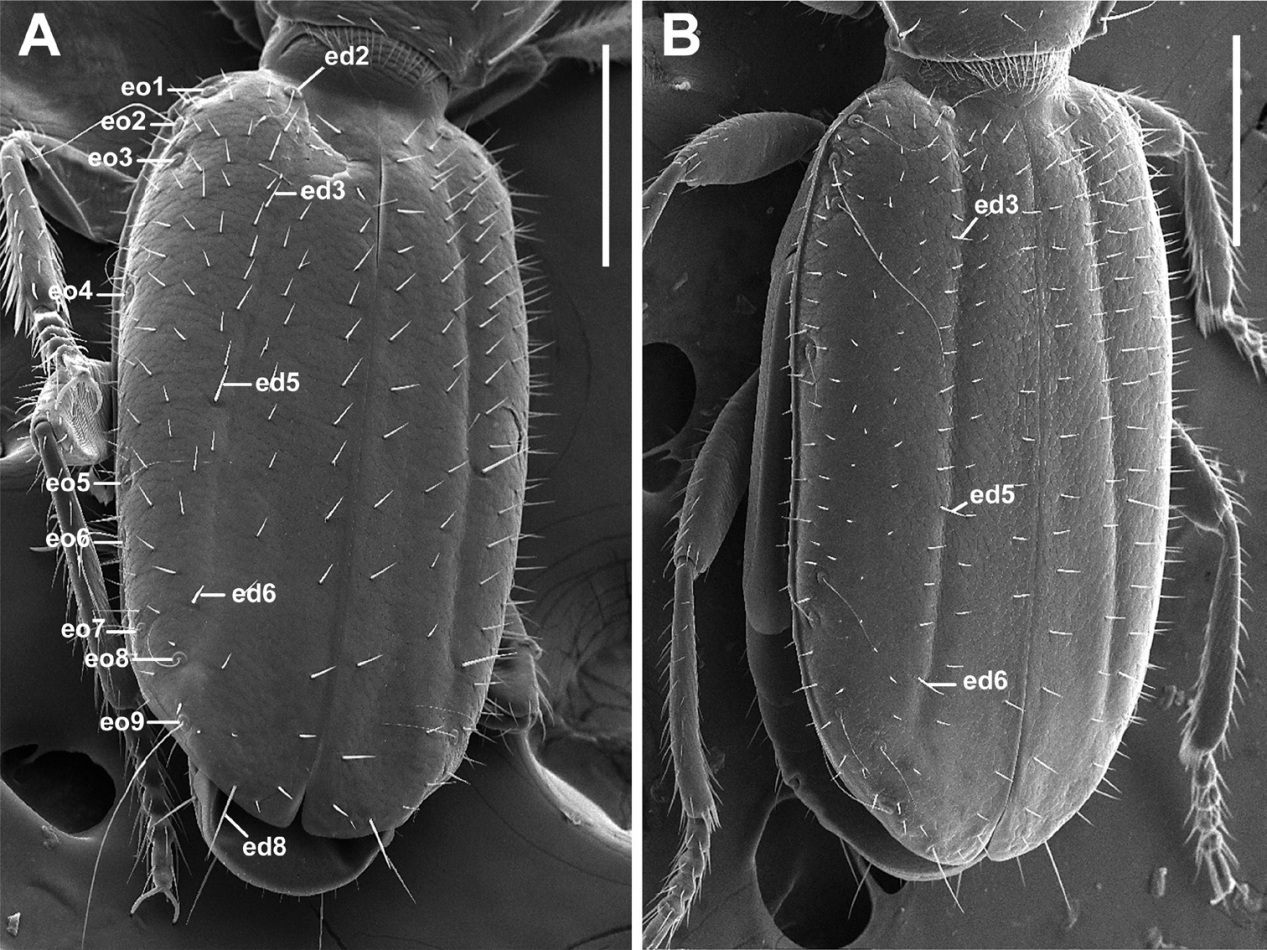
SEM illustrations of chaetotaxy of elytra, dorso-lateral aspect, of *Pelodiaetus* species. **A***P.sulcatipennis***B***P.nunni*. Abbreviations: ed2 – scutellar seta; ed3 – 1^st^ discal seta; ed5 – 2^nd^ discal seta; ed6 – 3^d^ discal seta; ed8 – apical seta; eo1–9 – setae 1–9 from the umbilical series. Scale bars 0.2 mm.

**Figure 5.**
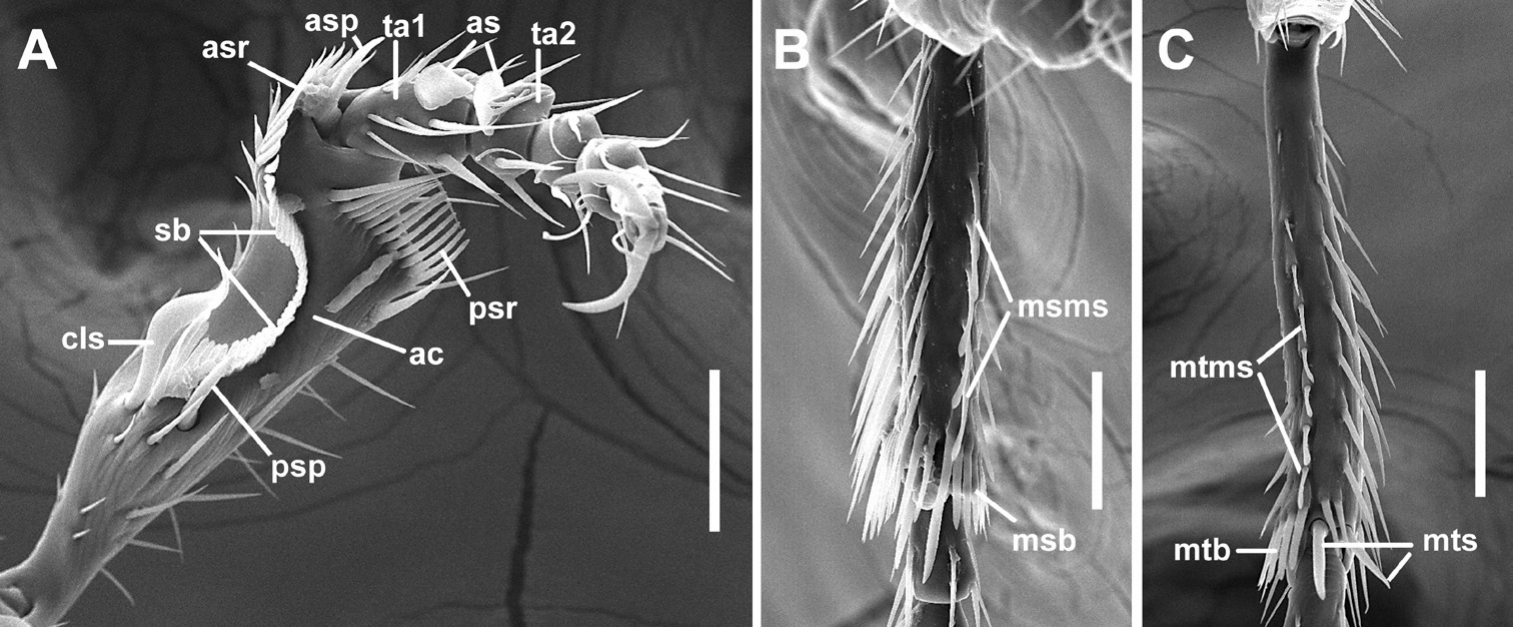
SEM illustrations of structural features of legs of *Pelodiaetussulcatipennis*, ventral aspects. **A** left protarsus and protibia **B** right mesotibia **C** left metatibia. Abbreviations: ac – antenna cleaner; as – adhesive setae; asp – anterior spur; asr – anterior setal row; cls – clip seta; msb – mesotibial brush; msms – mesotibial modified seta; mtb – metatibial brush; mtms – metatibial modified seta; mts – metatibial spur; psp – posterior spur; psr – posterior setal row; sb – setal band; ta1–ta2 – tarsomeres 1–2. Scale bars: 0.05 mm.

***Legs*** (Fig. 5). Legs of moderate length, not elongated. Prothoracic legs of males with first 2 tarsomeres (ta1–2) moderately dilated apico-laterally with one row of oval articulo-setae (as) (Stork 1980) on the ventral surface. Protibiae (Fig. 5A) with antenna cleaner (ac) of type B (Hlavac 1971), with both anterior (asr) and posterior (psr) apical setal rows long and with concave apico-lateral notch. Length of anterior spur (asp) slightly smaller than length of 1^st^ tarsomere (ta1). Profemora moderately swollen. Mesotibiae (Fig. 5B) with one long row of modified ventral setae (msms) at apical half, two terminal spurs and tibial brush (msb). Metafemora unmodified, metatibiae (Fig. 5C) with one row of modified ventral setae (mtms) in apical half, with two terminal spurs (mts) and tibial brush (mtb). Tarsi pentamerous, 1^st^ and 5^th^ tarsomeres are the longest, 2^nd^–4^th^ tarsomeres of equal length on the tarsi of all legs, 1^st^ tarsomere shorter than combined length of 2^nd^–4^th^ tarsomeres. Tarsal claws simple, untoothed.

***Abdominal ventrites.*** Five visible abdominal ventrites: 2^nd^ ventrite longest, 1.7–2.0 times longer than 3^rd^ or 4^th^, 3^rd^ and 4^th^ equal in length; the last, 5^th^, 1.6–1.8 times longer than 4^th^. Intercoxal process of 2^nd^ ventrite of moderate width, constricted anteriorly, subparallel before blunt apex (Fig. 3A, ipa).

***Male genitalia*** (Fig. 7). Median lobe of aedeagus anopic, elongate, slightly twisted and moderately arcuate. Apex of median lobe unmodified, only slightly enlarged in dorsal view. Internal sac with weakly sclerotized copulatory sclerites represented by flagellum-like structures combined at their basal or apical parts with small sclerotized plates of various size (ds). Ostial fields or spines of internal sac absent. Parameres bi-setose. Left paramere large and broad, evenly tapered to apex, right paramere moderately long. Ring sclerite ovoid, conically tapered apically with slightly elongated, triangular, handle-like extension.

***Female internal genitalia.*** Gonocoxite 1 asetose (Fig. 3B, gc1). Gonocoxite 2 falciform (gc2), 1.8–2.1 times longer than its basal width, moderately curved, with two ensiform (es) and one apical nematiform (ns) setae. Laterotergite (lt) with six or seven setae. Spermatheca (sp) small, weakly sclerotized (Fig. 3B, 7DH), of bean-shape. Length of spermathecal gland (sg) much greater than length of spermatheca. Spermathecal duct (sd) very long.

**Included taxa.** The genus comprises two species: *P.sulcatipennis* Jeannel, and *P.nunni* sp. nov.

**Geographic distribution.** The species of *Pelodiaetus* are known from the lowlands of three regions of the South Island of New Zealand (Fig. 8): Canterbury, Otago, and Southland. In Otago representatives of the genus deeply penetrate inland along the Clutha River valley.

**Habitat.** According to the label information, all specimens of *Pelodiaetus* were collected from washed soil samples, except one specimen, which was collected under a stone after rain. Collections were made in a vast spectrum of habitats: from improved pasture and tussocks to conifer (kahikatea and podocarp) and broadleaf (*Neopanax* and beech) forests. Beetles were collected during most months of the year, except February, July, and August.

**Relationships.** Morphologically, the closest relative of *Pelodiaetus* among the New Zealand anillines is *Pelodiaetodes*, the two genera together forming a distinct New Zealand lineage of Anillini. Both genera share developed fronto-lateral carinae, distinct pronotal posterior angles, a dilated prosternal process, nine setae in the elytral umbilical series of pores, longitudinal elytral grooves, and are distinguished by the combination of these characters from any other New Zealand Anillina. Compared with overseas Anillini it seems reasonable to group the members of *Pelodiaetus* with other anillines having grooved elytra, including the Australian *Illaphanus* Macleay and the Madagascan *Bulirschia* Giachino, *Malagasytyphlus* Giachino, and *Malagasydipnus* Giachino (Giachino 2005, 2008). Based on similarity between New Zealand *Pelodiaetus* and Australian *Illaphanus*, Jeannel (1937: 276–277) postulated that both genera had a common ancestor, and their ancestral stock inhabited East Gondwana at the time when it included modern territories of Australia, Tasmania, and, partly, New Zealand. I agree with Jeannel’s opinion and consider the representatives of the Australian genus *Illaphanus* as the sister-taxon to the members of the New Zealand *Pelodiaetus*-lineage.

##### A key for identification of adult representatives of *Pelodiaetus* from New Zealand

**Table:** 

1	Eyes lacking (Fig. 6). Posterior angles of pronotum distinct, projected outwards, without denticle anterior to them (Fig. 2A, B). Prosternal process dilating to blunt apex (Fig. 2C, D). Elytra with longitudinal grooves and uneven discal setae: 1^st^ discal seta not distinguishable from surrounding vestiture, noticeably shorter than distinct 2^nd^ and 3^d^ discal setae, which are clearly visible (Fig. 4). Umbilicate series of nine pores (Fig. 4)	**2**
–	Other combination of characters	**other genera of New Zealand Anillini**
2	Male with distal sclerites (ds) of internal sac long (Fig. 7A), formed as a long flagellum with strongly sclerotized compact basal area. Females with spermatheca moderately elongated, > 2.5 times longer than wide (Fig. 7D). Beetles from the Port Hills Range (Fig. 8, white circles), of coastal central-eastern Canterbury	***P.nunni* sp. nov.**
–	Males with distal sclerites (ds) of internal sac short (Fig. 7E), not longer than a half of the length of median lobe; sclerites without compact basal congestion. Females with spermatheca slightly elongated, <2.0 times longer than wide (Fig. 7H). Beetles from Stewart Island, Southland, Otago and foothills of southern Canterbury (Fig. 8, yellow circles)	***P.sulcatipennis* Jeannel**

###### Pelodiaetus nunni sp. nov.

Taxon classificationAnimaliaColeopteraCarabidae

D620588B-0C91-57CE-B250-51D8ED54EAD7

http://zoobank.org/FEFE7294-B8BE-406B-89E8-6D98B26F6D62

**Figure 6.**
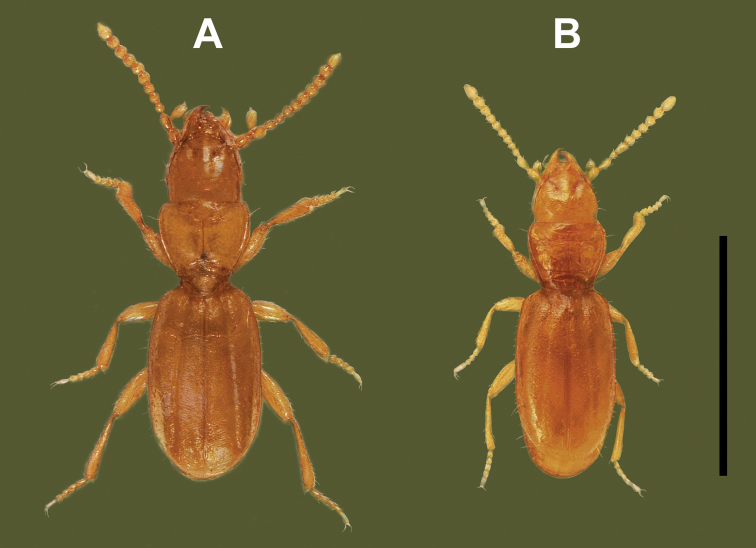
Digital images of habitus of *Pelodiaetus* species, dorsal aspect. **A***P.sulcatipennis* (NZ, Otago, Outram) **B***P.nunni* (NZ, Canterbury, Ahuriri Scenic Reserve). Scale bar: 1.0 mm.

**Figure 7.**
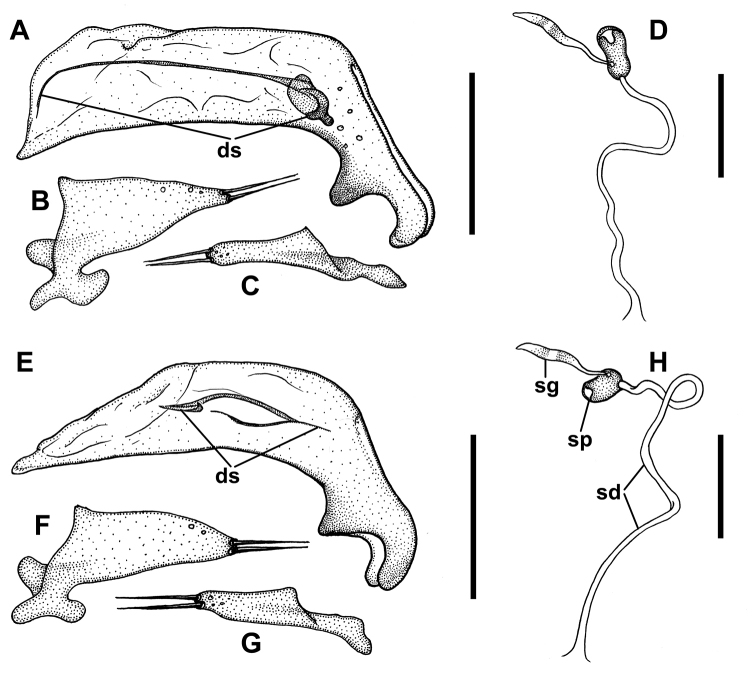
Line drawings of male genitalia and female spermathecae of *Pelodiaetus* species. *P.nunni* (NZ, Canterbury, Ahuriri Scenic Preserve): **A** median lobe, right lateral aspect **B** left paramere, left lateral aspect **C** right paramere, right lateral aspect **D** spermatheca. *P.sulcatipennis* (NZ, Otago, Outram): **E** median lobe, right lateral aspect **F** left paramere, left lateral aspect **G** right paramere, right lateral aspect **H** spermatheca. Abbreviations: ds – dorsal sclerites; sd – spermathecal duct; sg – spermathecal gland; sp – spermatheca. Scale bars: 0.1 mm (**A, B, C, E, F, G**); 0.05 mm (**D, H**).

**Figure 8.**
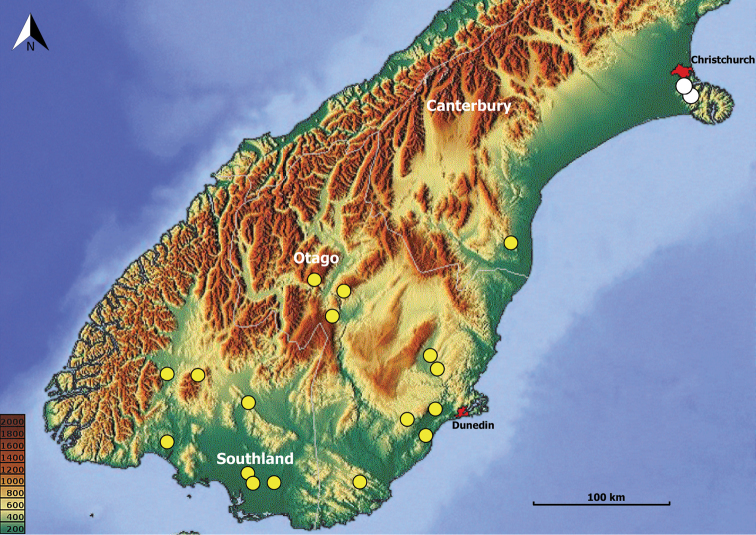
Map of southern half of South Island, New Zealand, showing positions of locality records for the species of *Pelodiaetus*: *P.nunni*, white circles; *P.sulcatipennis*, yellow circles. Elevation scale bars are given in meters.

Figs 1B, D , 2B, D , 4B , 6B , 7A-D , 8

####### Type material.

HOLOTYPE, male, in NZAC, labeled: / New Zealand MC Ahuriri SR Port Hills 31 May 08 / Washed soil sample, broad-leaf forest / NZMS 260 M36: 797303 455m /.

PARATYPES (7 specimens, in NZAC, JTN), 4 males and 2 females labeled same as holotype; 1 female labeled: / New Zealand MC Ahuriri SR Port Hills 7 Apr 07 / Washed soil sample, broad-leaf forest /.

####### Specific epithet.

The specific epithet is a Latinized eponym in the genitive case, and is based on the surname of John T. Nunn, the collector of this species.

####### Type locality.

New Zealand, South Island, Canterbury, Port Hills Range.

####### Recognition.

Adults of this species (Fig. 6B) are practically indistinguishable from the adults of *P.sulcatipennis* (Fig. 6A) and are distinguished from the latter by the structure of male and female genitalia.

####### Description.

With character states of the genus as summarized above.

***Size.*** Small to medium for genus (SBL range 1.25–1.32 mm, mean 1.30 ± 0.028 mm, n = 6).

***Habitus.*** Body form subdepressed, subparallel, moderately elongate (WE/SBL 0.33 ± 0.012), head comparatively wide for genus (WH/WPm 0.84 ± 0.011), pronotum of moderate width in comparison to elytra (WPm/WE 0.83 ± 0.045).

***Color.*** Body color rufo-testaceous, appendages testaceous.

***Prothorax.*** Pronotum moderately long (LP/LE 0.41 ± 0.011) and comparatively elongate (WPm/LP 1.19 ± 0.049), with lateral margins arcuately constricted posteriorly (WPm/WPp 1.48 ± 0.059). Width between anterior angles slightly greater than between posterior angles (WPa/WPp 1.21 ± 0.080).

***Elytra.*** Slightly depressed along suture, comparatively long (LE/SBL 0.57 ± 0.004) and moderately narrow (WE/LE 0.58 ± 0.019). Lateral margins slightly divergent at basal third, subparallel at middle and evenly rounded to apex in apical third.

***Male genitalia.*** Median lobe (Fig. 7A) with almost straight ventral margin and short apex with tapering tip. Sclerites of internal sac flagelliform, long, almost equal the length between apical and basal orifices, weakly sclerotized except basal enlargement.

***Female internal genitalia.*** Spermatheca weakly sclerotized, moderately elongate (Fig. 7D). Spermathecal duct long without coils. Attachments of spermathecal duct and gland to spermatheca close together.

####### Geographic distribution.

This species is known from the coastal Mid Canterbury area of Crosby et al. (1998), where its distribution is limited by Port Hills Range (Fig. 8, white circles).

####### Habitat.

Specimens were collected from soil in a broadleaf forest.

####### Relationships.

Based on the structure of male genitalia and spermatheca *P.nunni* is postulated to be the sister, more derived taxon of *P.sulcatipennis*.

###### Pelodiaetus sulcatipennis

Taxon classificationAnimaliaColeopteraCarabidae

Jeannel

C1B6AF79-563C-50C8-A5E1-049E0FD7DBAF

Pelodiaetus sulcatipennis Jeannel, 1937: 277 (original description); Jeannel 1963 (figures, key, distribution); Moore 1980 (key, distribution).
Pelodiaetus lewisi Jeannel, 1937: 277 (original description), **syn. nov.**; Jeannel 1963 (key, distribution); Moore 1980 (key, distribution).

####### Investigated material.

Stewart Island: Ulva Island Nov-Dec 2003 /coastal forest litter (5 specimens).

Southland: Tussock Creek Forest Hill Res 1 Sep 07 / Washed soil sample. Wet Podocarp/broadleaf forest (4 specimens); New Zealand SL Forest Hill Res nr Edendale 1 Sep 07 / Washed soil sample. Kahikitea forest (3 specimens) ; New Zealand SL Alton Burn Tuatapere SR 16 Nov 08 / Washed soil sample, totara/beech forest / NZMS 260 D45: 992419 38m (4 specimens) ; New Zealand SL Tuatapere Domain Tuatapere SR 16 Nov 08 / Washed soil sample, beech forest / NZMS 260 D45: 993403 30m (1 specimen) ; New Zealand SL Tuatapere Scenic Reserve at Tuatapere Domain, 30m J.Nunn 16 Nov 08 / Molecular voucher # 104 Sokolov I.M 2009 (1 specimen); New Zealand SL Tuatapere Scenic Reserve at Tuatapere Domain, 30m J.Nunn 16 Nov 08 / Molecular voucher # 107 Sokolov I.M 2009 (1 specimen); New Zealand SL Bog Burn Taringatura Forest 2 Jan 09 / Washed soil sample, beech forest / NZMS 260 E45: 397596 250m (1 specimen); New Zealand SL Dunsdale Stream, Hokonui Hills, 110m, washed soil sample J.Nunn 26 April 08 / Molecular voucher # 100 Sokolov I.M 2009 (1 specimen); New Zealand MC Pudding Hill Reserve Mt. Hutt 700m J.Nunn 25 Oct 08 / Molecular voucher # 105 Sokolov I.M 2009 (1 specimen); SL Chloris Pass, Catlins 17 Jun 06 (7 specimens); Rakahouka 2 Sep 07 /Neaopanax forest (2 specimens).

Dunedin: Woodside Glen, Outram 18 Nov 06 (3 specimens); Sutton, Salt Lake 21 Jun 09 / tussock and improved pasture (2 specimens); Picnic Gulley, Taieri Mouth 18 May 06 / under stone after rain (29 specimens); New Zealand DN Start of Government Tck Waipori Valley / Washed soil sample 17 Dec 06 (1 specimen).

Central Otago: Logan Burn 900m 13 Dec 1982 / ex. *Oreoboluspectinatus* (3 specimens); Cromwell, Kawarau Gorge, Roaring Meg 500m 12 Mar 1959 (2 specimens); Garden V., Raggedy Range 10 Sept 68 / Raoulia (1 specimen); Alexandra Hills, the Knobbies 10 Sept 68 / Raoulia (1 specimen).

South Canterbury: Guns Bush Waimate 23 Dec 06 / Washed soil sample, broadleaf forest (1 female).

####### Discussion.

As mentioned above, the genus *Pelodiaetus* was established by Jeannel (1937) for two species: *P.sulcatipennis* and *P.lewisi*. Both species were collected together in Dunedin, Otago, by G Lewis, and, according to Jeannel (1937, 1963), they can be distinguished from each other by the shape of the pronotum and elytra. In their descriptions, Jeannel did not provide comparison of the genitalia of the two species, and obviously did not make the necessary measurements for comparison of above-mentioned body parts. He simply restricted description to two couplets of a key with very general diagnoses: a more transverse and rounded pronotum with shorter and more convex elytra for *P.sulcatipennis*, and a correspondingly less transverse and rounded pronotum, and narrower and more elongated elytra, for *P.lewisi*. This short and very general comparison, together with the same type locality, made the validity of two forms questionable. Later, Moore (1980), reviewing the New Zealand Anillini, paid attention to the presence of intermediates in the body shape between the two species and stated that both names may belong to extremes of the same species, but did not formally synonymize these taxa because of the scarcity of the available material.

**Figure 9.**
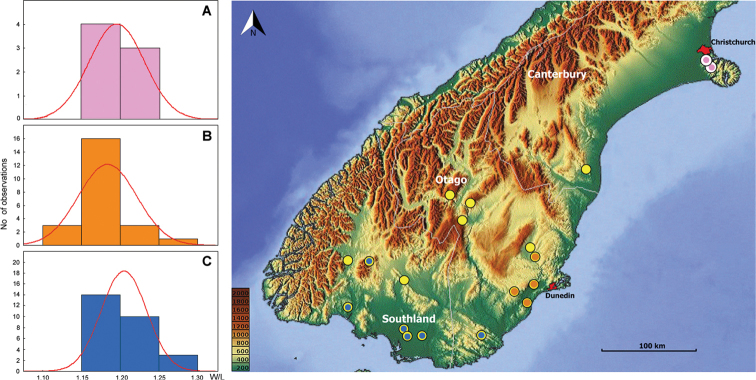
Variation in proportions of pronotum among different taxa and populations of *Pelodiaetus*. **A** – *P.nunni*; **B** – *P.sulcatipennis*, mid-eastern populations; **C** – *P.sulcatipennis*, south-western populations. Map of southern half of South Island, New Zealand, shows localities from where specimens were measured. Each species location dot (yellow or white) is identical to Fig. 8 but they are additionally marked with the appropriate bar graph color: pink, orange, and blue. X-axis values represent W/L – ratio of maximal width to length along the midline of pronotum. Red curve – expected normal distribution with sample mean and standard deviation. Elevation scale bars are given in meters.

Preparing this taxonomic review, I had an opportunity to investigate many more representatives of the genus. For analysis of the variation in body part proportions, measurements of 57 members of the genus were completed, including seven specimens of *P.nunni* and 50 specimens of *P.sulcatipennis*. Because of the wide range of *P.sulcatipennis*, the aim of this investigation was to compare main body ratios between the northeastern and southwestern parts of its range. All investigated ratios showed no difference between the two populations of *P.sulcatipennis*, as well as between the latter’s populations and the representatives of *P.nunni.* At the same time, many ratios showed rather high interspecies variability; this was especially true for W/L – ratio of maximal width to length along the midline of the pronotum, one of the characters used by Jeannel for distinguishing his species. According to the obtained data, the variation in the proportions of the pronotum (Fig. 9) is similar across different species of *Pelodiaetus* (T-test for independent groups, p = 0.693, n = 7 for *P.nunni*, and n = 50 for *P.sulcatipennis*) and between different populations of *P.sulcatipennis* (T-test for independent groups, p = 0.095, n = 23 for mid-eastern, and n = 27 for south-western populations). The only difference between populations of *P.sulcatipennis* can be seen in the light shift towards the prevalence of specimens with a slightly more transverse pronotum in the southwestern part of range. However, this deviation lies within the error range and is not statistically significant (see above). The type locality of *P.sulcatipennis* and *P.lewisi* (Dunedin, Otago) is located exactly within the range of the mid-eastern population, thus supporting the point of view of Moore that both Jeannel’s species may belong to extremes of a single species. In addition, in investigating male genitalia, I was unable to find any constant difference in the shape of the median lobe and in the armature of the internal sac. On the basis of this evidence, *P.sulcatipennis* and *P.lewisi* should be considered as variations of the same species and be synonymized under the name of *Pelodiaetussulcatipennis* Jeannel.

###### Pelodiaetus

Taxon classificationAnimaliaColeopteraCarabidae

sp.

F7A079F5-EE6B-5567-BC35-4965B87990F4

####### Material.

New Zealand: South Island, Nelson, Brightwater, Torpedo Pipe, May 1974 (1 female).

This locality lies far outside the range of *Pelodiaetus*. To confirm the locality and to clarify the taxonomic status of a local population more material including males is needed. At present the status of this specimen is uncertain and it is not considered in the following discussion.

## Discussion

### Range of the genus in the light of geological data and taxon-area concordant relationships

The range of *Pelodiaetus* species stretches across three regions of the South Island of New Zealand (Fig. 8) from Canterbury in the north through Otago and down to Southland and Stewart Island in the south. The members of the genus inhabit almost all the territory of the coastal lowlands, and in Otago penetrate deeply inland along the Clutha River valley. This rather large territory harbors only two species, one of which is endemic to the Christchurch area and the other of which occupies the rest of the genus range. Morphologically, the two species are almost identical and can be distinguished from each other only after dissection and genitalia investigation, suggesting that diversification between species took place relatively recently.

Geologically, the range of the genus is associated with old terranes composed of Paleozoic to Early Cretaceous volcanics or sediments (Mortimer 2004, Michaux 2009). The range of *P.sulcatipennis* occupies areas on older island arc-derived and oceanic terranes, namely, the Brook Street, the Murihuku, the Dun Mountain-Maitai, and part of the younger Rakaia Terrane (Wandres and Bradshaw 2005, Liebherr et al. 2011, Cooper and Ireland 2013). The range of the presumably more recently derived *P.nunni* is associated with the younger Rakaia Terrane only (Wandres and Bradshaw 2005, Liebherr et al. 2011, Cooper and Ireland 2013).

**Figure 10.**
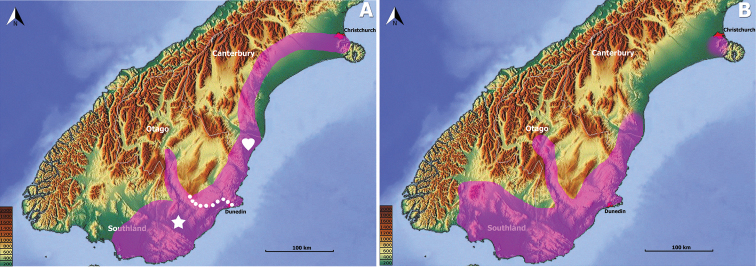
Map of southern half of South Island, New Zealand, showing ranges of subterranean non-montane Anillina. **A** – *Zeanillus* (combined ranges of subgenera *Zeanillus* s. str. (heart) and *Brounanillus* (star), white dots represent approximate boundary between taxon ranges); **B** – *Pelodiaetus*. Elevation scale bars are given in meters.

Search of concordant taxon-area relationships reveals that non-montane representatives of another anilline endogean taxon, the genus *Zeanillus*, demonstrate almost identical distribution to the members of the genus *Pelodiaetus* (Fig. 10). Indeed, the range of *Pelodiaetus* in its main contours repeats the range of the monotypic subgenus Brounanillus Sokolov (Fig. 10A, star), but, in comparison to the range of the latter, demonstrates evident features of northward and westward expansions. Interestingly, northward expansion of the range of *Pelodiaetus* corresponds to the range of the nominotypical subgenus Zeanillus (Fig. 10A, heart), a group of closely related species inhabiting coastal foothills of southern and central Canterbury. Similar to the members of *Pelodiaetus*, in the Christchurch area the members of *Zeanillus* s. str. are represented by their own endemic species *Z.phyllobius* (Broun). The similarity in general distribution and particular species ranges between the members of *Pelodiaetus* and *Zeanillus* may point to their at least partly shared evolutionary history in the South Island.

### Notes on the evolutionary history of *Pelodiaetus*

In the history of Zealandia (Mortimer et al. 2017), the remnant of which is New Zealand (Heads 2016), there was a critical time point for the local biota known as the Oligocene “drowning” (Haq et al. 1987, Stöckler et al. 2002). Depending on the scenario of maximal marine transgression, i.e., on how much the land of Zealandia was above sea level at that time, there exist two concepts of the shaping of modern New Zealand biota, called the “dispersal” and “vicariance” models (Heads 2016). One group of scientists assumes that complete submergence of subaerial lands with subsequent elimination of terrestrial biota took place and believes that all modern lineages arrived in New Zealand after the Oligocene (dispersal model) (Waters and Craw 2006, Landis et al. 2008, Wallis and Trewick 2009). Another group supports the idea that during maximum marine transgression, Zealandia was only reduced to a certain number of low-lying islands on which the Gondwanan-aged relict taxa survived drowning (vicariance model) (Lee et al. 2001, Knapp et al. 2007, Giribet and Boyer 2010, Conran et al. 2014). The second scenario implies that reduction in size of subaerial lands caused corresponding reduction in species diversity, and that these islands remained isolated for tens or hundreds of thousands of years, thus providing enhanced opportunities for geographic speciation (Cooper and Cooper 1995).

Contemporary distribution of the members of the genus *Pelodiaetus* and its relatives seems to fit better the vicariance model, because it is likely that their ancestors persisted on Zealandia after it’s rifting off and drifting away from Australia. The distribution of putative overseas relatives of *Pelodiaetus* serves as an indirect support for this assumption. As mentioned under the genus description, the members of *Pelodiaetus* are very similar to the members of the primarily Australian genus *Illaphanus* that comprises 29 species of small to medium-large (0.86–1.74 mm) beetles. Besides the Australian mainland, representatives of this genus are known from Tasmania and from the Lord Howe and Norfolk Islands (Giachino 2005). All *Illaphanus* species have grooved elytra, no denticle in front of the posterolateral angles of the pronotum, and, with some exceptions, are lacking the mental tooth in the labial complex. The state of the latter character in most cases distinguishes the members of *Illaphanus* from New Zealand representatives of *Pelodiaetus*, which are characterized by the blunt mental tooth. However, the state of the mental tooth varies within *Illaphanus*, and some species of *Illaphanus* with the developed mental tooth are almost indistinguishable from the members of *Pelodiaetus*. Notably, a small *Illaphanus* with the mental tooth, *Ill.norfolkensis* Giachino, occurs on Norfolk Island (Giachino 2005), which is famous for its endemic fauna of considerably older age than the island itself (Heads 2011). It has been hypothesized that endemic taxa surviving today on the island evolved on other former islands existing in the vicinity on the Norfolk Ridge, the strip of continental crust that extends from New Zealand to New Caledonia. During the breakup of East Gondwana, the Norfolk Ridge together with the Lord Howe Rise and the rest of Zealandia drifted away from Australia (Heads 2016). Hence, the occurrence of *Illaphanus* species on Norfolk and Lord Howe Islands supports the idea that *Pelodiaetus*-like forms might have persisted on the territory that split from East Gondwana as the Zealandia continent ca. 84 Ma ago (Mortimer et al. 2019).

One more piece of evidence of the persistence of the representatives of the *Pelodiaetus* lineage in Zealandia is the geographical distribution of the members of *Pelodiaetodes*, presumably the sister-taxon to *Pelodiaetus* (Sokolov 2015). Externally, besides their large size, the members of *Pelodiaetodes* can be distinguished from the members of *Pelodiaetus* only by the well-developed denticle anterior to the posterolateral angles of the pronotum. This character is not known among the members of *Illaphanus* and other genera with grooved elytra and can be considered as an autapomorphy of *Pelodiaetodes*. The genus comprises two morphologically distinct subgenera; its modern distribution is disjunct and limited by the Northland Allochthon (Bradshaw 2004) on the North Island (nominotypical subgenus, four species) and one locality in the Oamaru region on the South Island (subgenus Monosetodes Sokolov, one species). Given that the lands of the Northland Allochthon were completely submerged during the late Oligocene and became subaerial only around the late- to mid-Miocene after subduction of the Pacific Plate under the Australian one (Bradshaw 2004), the later arrival of the members of *Pelodiaetodes* to the North Island can be assumed. The Oamaru region remained submerged from the Late Cretaceous to the early Oligocene, but some lands of this region were likely exposed during the Oligocene “drowning” from the late Oligocene to the early Miocene (Thompson et al. 2014), which is supportive of the idea of the late arrival of the members of *Pelodiaetodes* to the Oamaru region as well. The absence of *Pelodiaetodes*-like forms in Australia but their presence as two morphologically distinct taxa in New Zealand suggests that the genus *Pelodiaetodes* arose after the East Gondwana breakup, but before New Zealand acquired its modern contour, i.e., somewhere on the Zealandia continent. Apparently, the members of *Pelodiaetodes* managed to survive different epochs of Zealandia inundations including the Oligocene “drowning,” and finally reach the places of their modern distribution.

Additionally, it is worth mentioning a strange ecological pattern of co-occurrences of the members of *Pelodiaetus* with other anillines. In the examined material, I came across four cases of syntopic co-occurrences of New Zealand anillines. Three of these cases were related to *Pelodiaetus* species: namely, *Pelodiaetusnunni* co-occurred with Z. (Zeanillus) phyllobius (Broun) at Ahuriri (Christchurch area, Canterbury), *P.sulcatipennis* with Z. (Zeanillus) nunni Sokolov at Woodside Glen (Dunedin area, Central Otago), *P.sulcatipennis* with Z. (Brounanillus) pallidus (Broun) at Picnic Gully (Taieri Mouth, South Otago), and one case related to its sister-taxon, where Z. (Nunnanillus) pellucidus Sokolov co-occurred with Pelodiaetodes (Monosetodes) nunni Sokolov at Glen Warren Reserve (Oamaru, North Otago). In these examples, the co-occurring species were represented by two contrasting morphotypes, a small (first place in the listing above) and a large one. In all cases but one, a small species of the *Pelodiaetus* lineage co-occurs with a large one from the unrelated clade, i.e., a small *Pelodiaetus* lives together with a large *Zeanillus*. However, in the fourth case, on the contrary, a small *Zeanillus* lives with a large *Pelodiaetodes*. Generally, miniaturization is one of the ways of speciation in the Anillini allowing species to occur syntopically and to exploit resources more effectively (Sokolov 2013, Andújar et al. 2017). Often these species are closely related and probably formed as a result of sympatric speciation, as was demonstrated for North American *Anillinuslescheni* Sokolov and Carlton and *A.stephani* Sokolov and Carlton (Sokolov et al. 2004), for some European *Typhlocharis* Dieck (Pérez-Gonzáles and Zaballos 2013), and for Mexican *Zapotecanillusoaxacanus* Sokolov and *Z.nanus* Sokolov (Sokolov 2013). In the case described in this paper, the co-occurrence of small and large species belonging to unrelated taxa demonstrates evolutionary adaptations of representatives of different genera to living together in one community, and also suggests intermixture of different faunas, reflecting the complicated evolutionary history of endogean biota on the Zealandia continent. Apparently, the evolution of the local anilline community was accompanied by the subsequent extinction and replacement of one taxon with another, either because of dramatic historical events or as a result of species competition, or as a combination of both.

Comparison of species composition in Australia and New Zealand helps to explain the origin of the South Island anilline faunas. In Australia, as many as 29 (85.3%) of 34 known species of Anillini belong to the genus *Illaphanus*, the sister-taxon to the *Pelodiaetus* lineage. The same dominance of representatives of the *Pelodiaetus* lineage can be seen on the North Island, where four species (80.0%) of five recorded for the island belong to *Pelodiaetodes*. At the same time, on the South Island, only three species (20.0%) of 15 anillines belong to the *Pelodiaetus* lineage. The significant decline in the number of species of the *Pelodiaetus* lineage on the South Island suggests that the Anillini faunas of the two islands may have different origins. The presence of species with Australian roots on the North Island is in concord with well-documented southeastern Australia-New Zealand connections (Heads 2014, 2016). By contrast, on the South Island, the fauna of Anillini has been shaped by the representatives of *Zeanillus*, whose ancestors supposedly inhabited a territory of the Campbell Plateau (Sokolov 2016). The geological data show that the Campbell Plateau together with Marie Byrd Land formed the West Antarctic margin, rather distant from the Australia/Pacific Plate margin of East Gondwana (Siddoway 2008, Heads 2014). Thus, the anilline community of the South Island could have resulted from an intermixture of ancient endogean faunas from different parts of East Gondwana and perhaps was dramatically modified by the Oligocene “drowning.” The possibility that different parts of East Gondwana could harbor different terrestrial faunas seems plausible. Several recent investigations have demonstrated examples of diversification of ancient terrestrial groups that had taken place prior to major geological events that split ancient supercontinents (San Mauro et al. 2005, Murienne et al. 2014, Heads 2014, Buckley et al. 2015).

## Supplementary Material

XML Treatment for Pelodiaetus

XML Treatment for Pelodiaetus nunni

XML Treatment for Pelodiaetus sulcatipennis

XML Treatment for Pelodiaetus
